# Navigating Uncertainty: A Case Study of Intrahepatic Cholestasis of Pregnancy
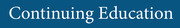


**DOI:** 10.1111/jmwh.13362

**Published:** 2022-04-04

**Authors:** Julie Blumenfeld, Kristin Koo

**Affiliations:** ^1^ Midwifery Program, Division of Advanced Nursing Practice Rutgers School of Nursing Newark New Jersey; ^2^ Capital Health Trenton New Jersey

**Keywords:** cholestasis, intrahepatic, health disparities, liver disease, pregnancy, pruritus

## Abstract

Intrahepatic cholestasis of pregnancy (ICP), the most common liver disorder of pregnancy, is associated with complications for both a pregnant person and their fetus. The underlying cause is not well understood. The pruritus associated with ICP is uncomfortable for pregnant people; however, the primary concern is the fetal risk. Fetal risks include preterm labor and birth and intrauterine fetal demise. This is particularly significant for certain populations because of the disparities in incidence of ICP; in the United States, it disproportionately affects Latinx people, the largest and fastest‐growing minority population. Diagnosis, monitoring, and treatment of ICP are vital to reduce discomfort from pruritis and avoid potential fetal demise. However, diagnosis and treatment are complicated by the lack of definitive diagnostic criteria, the frequent delay in laboratory analysis, and the cost of treatment. This case report aims to improve midwives’ familiarity with ICP and discusses the epidemiology, risk factors, presentation, diagnostic criteria, and available management strategies for this disease as well as the importance of anticipatory guidance regarding increased lifetime risk of ICP in future pregnancies and hepatobiliary disease. Additionally, it discusses the challenges involved in diagnosis and access to treatment. Prompt diagnosis and initiation of treatment may reduce fetal morbidity and mortality.

## CASE SUMMARY

*M.C. is a 36‐year‐old gravida 4, para 3003 K'iche’ woman from Guatemala who presented for her first prenatal visit at 25 weeks’ gestation. Her history includes 3 prior term pregnancies, the third of which in 2012 was complicated by intrahepatic cholestasis of pregnancy (ICP). She had a history of a cholecystectomy in 2019. The remainder of her health history and review of systems was noncontributory*.

*At her routine prenatal visit at 31 weeks’ gestation M.C. reported to the midwife that she was intermittently itchy and attributed it to dry skin. She was using an oatmeal salve with good relief. Upon examination she had no rash. Because of her prior pregnancy history and her itching the midwife recommended fasting total bile acids, alanine transaminase (ALT) and aspartate transaminase (AST) to check M.C. for possible ICP. The laboratory results were as follows: ALT 30 U/L, AST 38 U/L, and total bile acids 4.5 μmol/L. These were reported as normal by the laboratory. The midwife provided reassurance and recommended continuing with routine prenatal care*.

*At a later visit at 35 weeks and 2 days’ gestation, M.C. reported increased itching, particularly at night. On examination, her legs were excoriated from scratching. Because of her worsening itching, her midwife recommended repeating the fasting total bile acids, ALT, and AST to again rule out ICP. A high suspicion of ICP was discussed with M.C., and a plan was made to reassess once the laboratory results were available. The liver function tests returned later that day: ALT 104 U/L and AST 63 U/L. Given her history, these abnormal results, and M.C.’s persistent itching, the midwife consulted with the onsite obstetrician‐gynecologist who agreed with the midwife's recommendation to initiate treatment with ursodiol (Actigall) 300 mg orally twice a day. Later that day M.C. contacted the midwife to verify the need for the medication, expressing her hesitation because of its cost. Despite meeting her state Medicaid's eligibility requirements, M.C. did not qualify for enrollment because of her immigration status. The midwife reviewed the rationale for the medication and suggested purchasing a one‐week supply, which could then be refilled based on the pending bile acid results. A link to an online coupon was provided for a discounted price for the medication*.

  

Continuing education (CE) is available for this article. To obtain CE online, please visit http://www.jmwhce.org. A CE form that includes the test questions is available in the print edition of this issue.

*Five days later M.C.’s midwife called to inform the patient that her elevated bile acids, 79 μmol/L, indicated that she met criteria for the diagnosis of ICP. Because of her unremitting pruritus and bile acids greater than 40 μmol/L, the midwife in collaboration with the consulting physician recommended an immediate induction of labor*.

*An induction of labor was scheduled for later that day. Because of M.C.’s gestational age of 36 weeks, upon her admission to the labor and birth unit the midwife and the consulting physician recommended the administration of antenatal corticosteroids for fetal lung maturity*.

*After discussion of options for induction of labor, M.C.’s midwife placed a cervical ripening balloon and initiated a Pitocin infusion. The balloon fell out 11 hours after placement, M.C.’s labor progressed, and she gave birth 5 hours later to a vigorous female newborn with Apgar scores of 8 and 9 at one and 5 minutes*.

*M.C. had a clinically uneventful postpartum course. She reported that her itching rapidly dissipated over the first 48 hours postpartum. M.C.’s midwife discussed with her that because of her diagnosis of ICP, she is at increased risk for recurrence of ICP in subsequent pregnancies. Additionally, she encouraged her to let her primary care provider know of her diagnosis as she is at increased risk for future hepatobiliary disease*.

Note: This case is a composite of elements from different patients.

## INTRODUCTION

ICP is the most common liver disorder specific to pregnancy and is associated with pregnancy and fetal complications.^1^ The underlying cause is poorly understood.^1^ Although the pruritus associated with ICP is uncomfortable for pregnant people, the primary concern is the risk to the fetus.^2^, ^3^ Fetal risks are notable and include increased meconium‐stained amniotic fluid, neonatal respiratory distress, preterm labor, preterm birth, and intrauterine fetal demise.^1^, ^4^, ^5^, ^6^ This is especially the case for certain populations, as there are disparities in incidence of ICP; in the United States, it disproportionately affects Latinx people.^7^, ^8^ In 2020 there were 62.1 million Latinx people in the United States, composing about 19% of the total population.^9^ Latinx individuals are the largest and fastest‐growing ethnic minority group in the United States. In 2020, 24% of the births in the United States were to Latinx individuals.^10^

Despite the poor fetal outcomes associated with the condition, there is a lack of consensus regarding diagnostic criteria and best practice to reduce perinatal morbidity.^8^, ^11^ In this article we aim to provide an overview of the epidemiology, pathophysiology, clinical presentation, diagnosis, treatment, and long‐term health implications of ICP to raise awareness of the condition and guide treatment of affected patients.

## EPIDEMIOLOGY

The prevalence of ICP varies widely based on geography.^1^, ^6^ Rates in Latin America have been reported to be in ranges as high as 4% to 22%, with the highest rates occurring in indigenous populations in Chile.^7^, ^8^ Estimates of prevalence in North America and Europe are lower, ranging from 0.4% to 1.0%, and primarily concentrated in Scandinavia; a large longitudinal study in Sweden showed rates of 0.3% to 0.5%.^1^, ^3^, ^12^ In a unique study of ICP in a predominantly Latinx population in California, Lee et al^8^ noted that the prevalence of ICP in their patient population was 5.6%, more than 10 to 100 times the reported rate in the general population in the United States. Overall, individuals of Indigenous American ancestry have higher rates of ICP when compared with those of European descent without Indigenous American ancestry.^7^

## PATHOPHYSIOLOGY

The disease process of ICP, a disorder of bile acids, is not well understood. The underlying cause is thought to be multifactorial with genetic, hormonal, and environmental components contributing to the disease process.^1^ There are numerous known risk factors (Table 1), including history of ICP in prior pregnancy, which increases risk in subsequent pregnancy as much as 90%.^13^ Individuals with family members who have had ICP are at increased risk, particularly if they are first‐degree relatives. Genetic predisposition has also been linked to specific gene mutations in populations with increased incidence of ICP in both Europe and South America.^1^ There is a hormonal element to the disease process; pregnant people with ICP lack symptoms both prior to pregnancy and postnatally.^1^ Additionally, there are individuals who develop cholestasis associated with use of oral contraceptives.^2^, ^6^ Although ICP may occur at any time, there is a seasonal variation to the disease process; it is more common in the winter months.^1^

**Table 1:** Risk Factors for Intrahepatic Cholestasis of Pregnancy

Risk Factors
Maternal age >35
History of ICP in a prior pregnancy
First‐degree relative with ICP
Preexisting liver pathology
Multiple gestation
Seasonal variation: winter

Abbreviation: ICP, intrahepatic cholestasis of pregnancy.

Sources: Pataia et al,^1^ Dixon and Williamson,^2^ and Tran et al.^6^

In a healthy person, cholesterol is used to synthesize bile acids in the liver, which are then stored in the gallbladder and released into the intestines to aid digestion.^2^ In ICP there is a buildup of bile acids in the liver, resulting in elevated serum bile acids. This is possibly due to alterations in the genes that regulate bile acid elimination and its subsequent movement into the circulation.^2^ Additionally, increased estrogen and progesterone in the third trimester are associated with impaired bile flow through the liver, causing bile to enter the bloodstream.^3^

Increased serum bile acids negatively affect fetal outcomes. Typically, the placenta protects the fetus by limiting exposure of toxic compounds such as bile acids. In ICP the placenta's protective mechanism that limits bile acid flow between pregnant person and fetus is impaired.^1^ As bile acids increase in the pregnant person so do those in the fetus, leading to poor fetal outcomes, such as meconium‐stained amniotic fluid, and stillbirth.^1^, ^4^, ^5^, ^6^ Additionally, increased bile acids damage the placenta, although the extent and nature of these changes are poorly understood. Researchers hypothesize that sudden death in utero may be due to vasoconstricted chorionic vessels of the placenta, leading to oxygen deprivation; an alternate theory posits that fetal arrhythmia secondary to increased bile acids leads to cardiac arrest.^13^

ICP also is associated with pregnancy complications including pruritis, preterm labor, gestational diabetes, and preeclampsia. Although in the past it was thought that there was a direct correlation between elevated bile acid levels and the severity of pruritis of ICP, this is not typically the case. Pruritis may precede bile acid elevations. Several hypotheses exist regarding the etiology of pruritis associated with ICP, including that bile acids may induce a signaling pathway in sensory nerves that could contribute to the itching and that progesterone sulphates may play a role.^2^ Although the exact etiology is unclear, research suggests that increased bile acids stimulate prostaglandin release and subsequent myometrial contractions, resulting in increased preterm labor.^1^, ^4^, ^5^, ^6^ This is particularly evident in individuals who genetically are at increased risk.^2^ There is an association with individuals with ICP and higher rates of glucose intolerance.^14^ There may also be an increased risk for preeclampsia in patients with ICP, particularly with bile acids greater than or equal to 40 μmol/L.^15^, ^16^

## CLINICAL PRESENTATION AND DIAGNOSIS

Typical presentation for ICP includes new onset of generalized body itching, often concentrated on the soles of hands and feet, and worse at night. Upon examination, there is no rash, although some patients may begin to have excoriations from excessive scratching. Pregnant people with ICP lack symptoms both prior to pregnancy and postnatally. The symptoms are isolated to pregnancy and typically resolve postpartum.^3^

Increased total bile acids is the biochemical marker found to correlate with perinatal risk and, as such, is the hallmark diagnosis for ICP.^4^ However, confirmatory diagnosis may be delayed because (1) pruritus and elevated liver function tests often precede increased serum bile acids and (2) special laboratory processing for total bile acids may take 4 to 14 days.^3^ When assessing for potential ICP, other hepatic disease and dermatoses of pregnancy should be excluded, including cholelithiasis, hepatitis, fatty liver, atopic eruption of pregnancy, and polymorphic eruption of pregnancy.^17^ Liver function tests are not required for ICP diagnosis, but transaminitis may heighten suspicion for ICP during the wait period for bile acid results.^3^ Conversely, normal range AST and ALT do not exclude ICP. Although the Society for Maternal‐Fetal Medicine (SMFM)^3^ notes that the variation between fasting and prandial bile acids is minimal and should not influence diagnosis, other authors suggest fasting bile acid levels are significantly lower than the nonfasting levels and should be used for diagnosis.^18^

Despite limited data to support a diagnostic criteria for ICP, bile acids greater than 10 μmol/L is commonly used.^3^ As bile acids increase, so does the level of risk for the fetus; total bile acids greater than or equal to 40 μmol/L categorizes a higher level of fetal risk.^2^, ^3^, ^6^, ^19^ In a systematic review Di Mascio et al^20^ found a 6.8% incidence of fetal and newborn death associated with bile acids greater than or equal to 100 μmol/L. They recommend that when levels are this high, perinatal care providers should consider immediate birth if the pregnancy is late preterm or term.

As bile acid levels can fluctuate during pregnancy, with higher levels closer to term, repeating testing in the presence of persistent or worsening symptoms will ensure appropriate diagnosis and avoid potential mismanagement. However, serial testing at regular intervals is not currently recommended by SMFM.^3^

## MANAGEMENT AND TREATMENT

Current guidelines regarding fetal and biochemical surveillance, pharmacologic management, and birth timing have been developed in an effort to reduce perinatal risk.

Goals of pharmacologic treatment focus on symptom relief and expedited birth to reduce risk to the fetus. Ursodeoxycholic acid, also referred to as ursodiol or brand name Actigall, is the current first‐line treatment for ICP and is considered safe for both the fetus and the pregnant person.^6^ Initial prescription can include 300 mg orally twice a day or 3 times a day or 500 mg orally twice a day. This dosage of 10 to 15 mg/kg per day divided into 2 or 3 doses can be increased to 21 mg/kg per day to further allay itching symptoms.^3^

Outside of pregnancy, ursodiol is used as a cholesterol‐reducing medication aimed at dissolving certain gallstones and normalizing and protecting liver metabolism.^21^ Ursodiol may improve the solubility of bile acids, facilitating their movement from the liver into the gallbladder and possibly diminishing their negative impact.^11^ However, it may take 1 to 2 weeks for symptom relief and 3 to 4 weeks for bile acids to decrease.^21^ Possible adverse effects include nausea, dizziness, and upset stomach.

There is a lack of conclusive evidence to substantiate ursodiol use to alleviate pruritis in pregnant people or diminish poor fetal outcomes. In a meta‐analysis of 12 randomized control trials including 662 participants, Kong et al^21^ concluded that when compared with control groups, ursodiol mitigated pruritus. However, in their larger and more recent review of 2 trials with 755 individuals, Walker et al^11^ found that although ursodiol likely diminishes the pruritis of ICP, the overall effect is minimal.

Ursodiol has not been shown to significantly improve fetal outcomes.^11^, ^22^ Kong et al^21^ reported that reduced bile acid concentration after several weeks of ursodiol use may reduce preterm labor and fetal distress. However, Walker et al,^11^ in their more recent review that included 7 studies with a total of 1008 individuals, concluded that there is no definitive evidence of ursodiol's efficacy to mitigate fetal distress or demise.

Outpatient fetal surveillance with serial nonstress tests may also be offered once ICP is diagnosed. However, the sudden and unpredictable course of cholestasis provides limited and possibly misleading reassurance from a reactive nonstress test, as fetal demise is sudden and may even occur within hours of monitoring.^3^, ^13^

Given the positive correlation between total bile acids and perinatal risk, induction of labor and early birth is recommended as the only intervention to prevent fetal demise. Puljic et al^23^ examined 1,604,386 low‐risk pregnancies to assess risk for stillbirth at any given gestational age. Risk of stillbirth was increased between 34 and 40 weeks’ gestation in those pregnancies found to be complicated by ICP. The authors concluded that facilitating birth at 36 weeks’ gestation lowers the risk of fetal death, compared with waiting for spontaneous labor and birth. In a 2021 systematic review, Di Mascio et al^20^ concluded that it is appropriate to consider birth at 35 to 36 weeks’ gestation if bile acids are greater than or equal to 100 μmol/L. Professional organizations provide recommendations of gestational age ranges for birth to lower the risk of fetal death (Table 2).

**Table 2:** Timing of Birth According to Total Bile Acids

Organization	Total Bile Acids μmol/L	Recommended Timing of Birth
American College of Obstetricians and Gynecologists	<100	36 0/7 to 39 0/7 wk or at diagnosis if diagnosed later; earlier based on laboratory and clinical circumstances
	≥100	36 0/7 wk or at diagnosis if diagnosed later; earlier based on laboratory and clinical circumstances
Society for Maternal‐Fetal Medicine	<40	36 0/7 to 39 0/7 wk; later end of the range is reasonable
	40‐99	36 0/7 to 39 0/7 wk; earlier end of range should be considered
	≥100	36 wk; consider birth at 34‐36 wk if intense and persistent pruritus unrelieved with medication, prior history of intrahepatic cholestasis of pregnancy with fetal demise at <36 wk gestation, or acute or preexisting liver disease with deteriorating liver function
Royal College of Obstetricians and Gynaecologists	Not specified	Discussion should take place regarding induction of labor after 37 0/7 wk; “severe biochemical abnormality” may more strongly compel intervention after 37 0/7 wk

Sources: Society for Maternal‐Fetal Medicine,^3^ American College of Obstetricians and Gynecologists,^24^ and Royal College of Obstetricians and Gynaecologists.^25^

Although both SMFM^3^ and the American College of Obstetricians and Gynecologists (ACOG)^24^ recommend birth at 36 weeks’ gestation when total bile acids reach 100 μmol/L, there is a lack of high‐quality evidence to support this.^3^ SMFM states that additional clinical circumstances, such as severe pruritus, history of ICP with stillbirth at less than 36 weeks’ gestation, or hepatic disease with worsening hepatic function, can warrant birth from 34 to 36 weeks.^3^ If total bile acids are less than 100 μmol/L, ACOG^24^ recommends a 3‐week birth window between 36 and 39 weeks’ gestation. SMFM recommends the same window; however, it suggests that a level of total bile acids of greater than or equal to 40 μmol/L favors birth earlier in the window.

Unlike ACOG and SMFM, the Royal College of Obstetricians and Gynaecologists^25^ does not specify a level of total bile acids to determine recommendations for birth timing. It calls for a discussion of birth after 37 weeks’ gestation, including outlining the risks and benefits of early intervention. The most recent publication was in 2011, with an update in 2014 stating that a revised guideline is deferred to a later date.

Early induction of birth for individuals with ICP does not guarantee fetal well‐being. A 2019 systematic review concluded that most people with ICP have bile acids between 10 to 99 μmol/L and that the risk of stillbirth may be comparable to those without ICP.^4^ However, the number of potential stillbirths due to ICP was likely not captured because of early intervention versus waiting for spontaneous labor. Iatrogenic risk of early induction should be included in patient counseling, such as cesarean birth and newborn admission to the intensive care unit. Ultimately, patient counseling and shared decision‐making are essential components of the management and treatment of ICP.

Typically, patients should be completely asymptomatic within 4 to 6 weeks postpartum. If not, they should be referred to a gastroenterologist for ongoing evaluation. Midwives should routinely provide education regarding future pregnancies and long‐term implications for both patient and offspring, such as risk of cardiovascular and hepatobiliary disease.^2^, ^26^

## CLINICAL IMPLICATIONS

The lack of consensus and infrequent updates for ICP guidelines in the past decade demonstrate the need for further comprehensive cholestasis research. Precise management and patient counseling regarding ICP are limited because of the unclear etiology. Furthermore, there is a scarcity of clinical studies, which in general are challenging to conduct in pregnant populations.

A close examination of the literature demonstrates a paucity of focus on the Latinx population despite evidence showing increased ICP risk to people of Latinx and Indigenous American ancestry. This disparity highlights the inadequate efforts aimed at understanding illnesses that differentially affect racial and ethnic minorities and their ongoing underrepresentation in clinical studies. At the least, an epidemiological survey of a disease of pregnancy that disproportionately affects the fastest‐growing population in the United States is overdue.

One immediately actionable improvement would be reducing the cost and improving access to ursodiol; the medication is cost‐prohibitive, particularly for individuals who lack health insurance with prescription coverage. Funding inclusive of pharmacologic treatments for all pregnant people regardless of immigration status is imperative. Predicting and preventing perinatal morbidity and mortality will continue to be challenging for perinatal care providers, as the consequences of ICP can be fatal.

## CONFLICT OF INTEREST

The authors have no conflicts of interest to disclose.
